# Exercise as a therapeutic option for acute kidney injury: mechanisms and considerations for the design of future clinical studies

**DOI:** 10.1186/s12882-020-02098-9

**Published:** 2020-10-23

**Authors:** Anam Asad, James O. Burton, Daniel S. March

**Affiliations:** 1 Department of Cardiovascular Sciences, University of Leicester, Leicester, UK; 2 NIHR Leicester Biomedical Research Centre, Leicester, UK; 3 School of Sport, Exercise and Health Sciences, Loughborough University, Loughborough, UK

**Keywords:** Acute kidney injury, Exercise, Rehabilitation, Kidney disease, Therapy

## Abstract

Acute kidney injury (AKI) is a known risk factor for chronic kidney disease (CKD) and end stage kidney disease (ESKD). The progression from AKI to CKD, despite being well recognised, is not completely understood, although sustained inflammation and fibrosis are implicated. A therapeutic intervention targeting the post AKI stage could reduce the progression to CKD, which has high levels of associated morbidity and mortality. Exercise has known anti-inflammatory effects with animal AKI models demonstrating its use as a therapeutic agent in abrogating renal injury. This suggests the use of an exercise rehabilitation programme in AKI patients following discharge could attenuate renal damage and improve long term patient outcomes. In this review article we outline considerations for future clinical studies of exercise in the AKI population.

## Background

Acute kidney injury (AKI) is defined as a sudden decline in kidney function demonstrated by either a rise in serum creatinine of at least 50% over baseline levels occurring within a 7-day time period, or a sudden decrease in urine output [1]. Globally, AKI occurs in 7–18% of patients in hospital, accounts for 50% of intensive care unit (ICU) admissions [2] and is responsible for 2 million deaths per year [3]. It is a complex condition with diffuse aetiologies resulting in both higher in-hospital and long-term mortality [4]. It has been demonstrated that there is an increased risk of chronic kidney disease (CKD) and end stage kidney disease (ESKD) following AKI survival with the risk increasing in proportion to the severity of AKI [5]. Despite being well recognised, the mechanism behind this increased risk is unknown, however AKI and CKD are now considered to be on a spectrum rather than as two separate entities [3]. This post-AKI state, whereby impaired renal function persists for > 7 days but < 90 days (therefore not fulfilling the diagnostic criteria of CKD) is referred to as acute kidney disease (AKD) [3]. This definition recognises the renal impairment that persists beyond the definition of AKI and prior to CKD. Furthermore, AKI can worsen underlying CKD [6]; over 30% of patients suffer repeated episodes of AKI [7] which in turn can further increase a patient’s CKD and ESKD risk. It is estimated that CKD occurs in approximately 25% of AKI survivors [8]. Not only is AKI a risk factor for CKD and ESKD but people who survive an episode are at an increased risk of developing non-renal conditions including congestive heart failure and cardiovascular disease [5]. The average hospital stay of an AKI admission is 16 days [9]. Therefore, AKI has a significant economic burden as it is estimated to cost the UK National Health Service (NHS) approximately £1.02 billion annually [9]. However, as many as one in five cases of AKI are thought to be preventable [10]. Therefore, with adequate measures to reduce AKI risk, effective monitoring of patients, and with the implementation of appropriate interventions, the NHS could save up to £200 million a year [9].

Current management strategies for treatment and prevention of AKI are limited. A number of trials [11–13] (which we have highlighted in Table 1), have unsuccessfully attempted to deliver interventions ranging from established pharmacological agents, furosemide, to more novel therapies such as alkaline phosphatase and mesenchymal stem cells. As a result, there is a need for new interventions and alternative strategies to be developed, to help promote renal recovery and improve outcomes following AKI [3, 19].

**Table 1:** Human Trials of therapies for either treatment or prevention of AKI

Author, year	Therapeutic Agent	Proposed Mechanism of therapeutic agent	AKI patients	Therapeutic Agent administration as treatment of prevention of AKI	Outcome
McCullough [11], 2016	Alpha Melanocyte Stimulating Hormone	Anti-inflammatory cytokine	Ischaemia/Reperfusion due to cardiac surgery	Prevention	↔ incidence of AKI ↔ NGAL ↔ Urine IL 18 ↔ Urine KIM-1
Swaminathan [12], 2018	Mesenchymal Stem Cells	Paracrine and endocrine effects	Ischaemia/Reperfusion due to cardiac surgery	Treatment	↔ time to recovery of kidney function ↔ dialysis requirement ↔ mortality
Bagshaw [13], 2017	Low Dose Furosemide	Reduces renal oxygen demand therefore alleviating oxidative stress	ICU admission with AKI	Treatment	↔ worsening of AKI ↔ kidney recovery ↔ RRT use ↔ mortality
Pickkers [14], 2018	Alkaline Phosphatase	Attenuation of inflammatory response	Sepsis	Treatment	↔ endogenous creatinine clearance ↑ RRT requirement ↔ kidney injury biomarkers
Kitzler et al. [15], 2012	Vitamin E and N-acetylcysteine	Vitamin E – antioxidative NAC – free radical scavenger	CKD stages 1–4 patients undergoing elective CT hence at risk of contrast induced AKI	Prevention	No patients developed contrast induced AKI ↔ change in creatinine clearance ↔ eGFR
Amendola [16], 2018	Goal- directed therapy	Maximises oxygen delivery and cardiac output to prevent tissue hypoxia. Correction of volume deficits and optimisation of haemodynamic status	Patients with early AKI in critical care	Treatment	↔ serum creatinine levels ↔ RRT requirement ↔ AKI beyond 72 h ↔ length of hospital stay
Ghaemian [17], 2018	Remote Ischaemic Preconditioning	Release of mediators which attenuate kidney damage following brief induction of ischaemia followed by reperfusion.	Patients with CKD undergoing coronary angiography or angioplasty therefore at risk of contrast induced AKI	Prevention	↔ serum cystatin C ↔ incidence of contrast induced AKI ↔ serum creatinine
Amini [18], 2018	Selenium, Vitamin C and N-Acetylcysteine	Antioxidants therefore prevent anti-oxidative stress	Patients undergoing off-pump coronary artery bypass graft surgery	Prevention	↔ incidence of AKI ↔ time of AKI occurrence ↔ severity of AKI ↔ duration of AKI ↔ length of hospital stay ↔ in hospital mortality

There is emerging evidence that utilising exercise as a therapeutic tool in the setting of AKI may hold promise; the primary aim of this review is to highlight this evidence. Secondary aims are to explore the mechanisms through which exercise may prove beneficial, and to explore the key considerations in designing future clinical trials in which the potential of exercise to promote recovery from an episode of AKI can be tested.

### Exercise and AKI

Exercise leads to a transient decrease in renal blood flow however with filtration fraction adjusting accordingly, normal renal function is maintained [20]. Two studies conducted in CKD populations [21, 22] have suggested improvements in renal function following 12-week exercise programmes, shown through significant improvements in eGFR [21, 22] and a significant decrease in proteinuria [22]. However, these results, although promising, must be interpreted with caution due to a number of shortcomings which include very small sample sizes (both are underpowered), inappropriate statistical analyses, and non-measurement of true renal function. Furthermore, in both instances the effect of exercise was shown in pre-dialysis CKD patients with no studies demonstrating the effects of exercise in the AKI setting. Despite this, the findings are consistent with other work in animal models [23–29] which have demonstrated an improvement in renal function following exercise in the AKI setting. Although the precise mechanism by which exercise, or increased levels of physical activity may protect against or even promote recovery following AKI in humans remains unclear, one mechanism which may be crucial is the well-reported anti-inflammatory effect of exercise. After the initial insult resulting in AKI, inflammation within the kidneys is the common denominator that propagates and sustains phases of renal injury [30], ultimately leading to scarring (fibrosis) and permanent loss of function. Exercise is known to have strong anti-inflammatory effects [31], the mechanisms for these effects include: the reduction in visceral fat which decreases secretion of pro-inflammatory adipokines [31]; the release of interleukin-6 (IL-6) from contracting skeletal muscle which increases levels of anti-inflammatory cytokines [32]; and the reduced production of proinflammatory cytokines secondary to the reduced expression of toll like receptors on monocytes and macrophages [33]. Despite these established mechanisms, limited work has been performed investigating the potential therapeutic effect of exercise in the setting of AKI.

### Methods of AKI induction in animal models

Animal models have used a range of methods to induce AKI, the most common being the administration of cisplatin, a known nephrotoxic drug [25–28] that is a widely used chemotherapy agent particularly effective in the treatment of solid tumours [34]. However, it can cause nephrotoxicity, which presents as deranged renal parameters including decreased GFR and increased serum creatinine [35]. The mechanism of cisplatin induced AKI is thought to be a combination of oxidative stress, inflammation and vascular insult to the kidneys [36]. Other methods of AKI induction in these animal exercise models, include gentamicin [24] and ischaemic reperfusion (IR) injury [23]. Whether the results seen in animal pre-clinical models can be repeated in humans has been discussed previously [37, 38]. The promising results of many therapeutics seen in animal AKI models [39–44], were unable to be repeated in AKI patients [11–18, 45]. This has been explained by numerous factors including the failure of pre-clinical studies to model AKI accurately. Pre-clinical studies often fail to account for the heterogeneity of the AKI population with factors such as co-morbidities, polypharmacy and severity of AKI influencing a patient’s response to therapy [37, 38] . A particular issue noted with cisplatin induced AKI animal models is that the induction of AKI is often through a single high dose cisplatin injection as opposed to lower doses over a longer period; a more clinically accurate method simulating cancer treatments. Despite the cisplatin models discussed below [25–28] which used a single high dose cisplatin injection, their clearly defined timings and dosages of the exercise administered allows for increased transferability of their results to humans, hence guiding future clinical studies. The effect of exercise in animal AKI models is discussed below, with potential mechanisms by which exercise attenuates renal damage being highlighted.

### Anti-inflammatory effects

A study by Miyagi et al. [25] investigated the role of exercise in cisplatin induced AKI and reported that a 6-week programme of exercise was protective against the apoptotic effects of cisplatin in the kidneys. However, the study was not able to elucidate the mechanism behind this as no difference was observed in B-cell lymphoma 2 (Bcl2) and Bcl-2 Associated X protein (BAX) following analysis. These anti-apoptotic and pro-apoptotic molecules respectively showed no differences amongst the exercise and sedentary groups despite it being postulated that the reduction in renal tissue cell death in the exercise group could be due to the increase in anti-apoptotic molecule Bcl2 compared with BAX. A decrease in BAX had also been previously shown to ameliorate cisplatin induced renal injury therefore protecting renal function [46]. The lack of effect on BAX [25] may be explained by the timing of measurement as during the later stages of apoptosis diffuse protein breakdown may affect its measurement [46].

Miyagi et al. [25] also demonstrated an increase in IL-6 following exercise, which was thought to have a negative feedback response to the AKI induced inflammation, contributing to the attenuation of renal damage. This is of particular interest since a well-known effect of exercise in humans is a transient increase in IL-6 [31, 47]. Exercise induced skeletal muscle contraction has shown to increase IL-6 levels [31, 47] which in turn blunt the increase in Tumour Necrosis Factor-alpha (TNF-a) levels after exposure to an inflammatory stimulus [48]. The increase in IL-6 and reduction in TNF-a levels following exercise in humans suggests potential therapeutic benefit of exercise in controlling low-grade inflammation, which can contribute to the progression of CKD.

In a follow up study [26] from the same group the anti-inflammatory effects of a 5-week programme of exercise were investigated. The study corroborated their previous results by showing reduced kidney injury in exercised cisplatin rats compared to their resting counterparts. This was shown through exercise blunting the increase in serum creatinine following cisplatin administration, and a reduction in T cell immunoglobulin mucin 1 (Kim-1) levels. Kim-1 plays an important role in regulating T cell response. Favourable effects on CD4+ T cells in the kidney draining lymph nodes were also observed. They reported that the suppression of CD4+ T cells by exercise resulted in reduced levels of TNF-a, a proinflammatory cytokine with a central role in cisplatin nephrotoxicity pathogenesis [49]. T-cells play an important role in the pathogenesis of AKI, and it has been suggested that interventions (such as exercise) that modulate activity of these cells, may ameliorate renal injury in patients [26]. The study also reported an increased expression of IL-6 in the exercise group. IL-6 is thought to have a protective role in cisplatin induced AKI [46, 50, 51], and it has been shown that IL-6 knock out mice have increased renal damage following cisplatin administration [51].

### Anti-fibrotic effects

A further cisplatin AKI animal study investigated the effect of a 4-week [27] exercise regimen, which was shown to be efficacious. The study demonstrated beneficial effects on circulating concentrations of urea, creatinine, and urinary markers of renal function. Furthermore, the study [27] showed evidence of histological improvements, evidenced by reductions in macrophage infiltration and IL-1B levels following exercise. The blunted macrophage response has implications for fibrosis since they are involved in releasing TFG-B, endothelin and angiotensin II, which are fibrogenic molecules. This study therefore demonstrates a potential anti-fibrotic mechanism by which exercise could reduce damage following AKI.

### Anti-oxidative effects

In addition to the anti-inflammatory [26] and anti-fibrotic [27] effects observed previously, exercise has been shown to have anti-oxidative effects [28] in a cisplatin induced AKI model. Rats subjected to an 8-week exercise regimen displayed blunting of malondialdehyde (MDA), a marker of oxidative stress [28]. This supports data from another study that showed reduced MDA levels in skeletal muscle and the liver following exercise [52]. It also suggests a more prolonged anti-oxidative effect of exercise through increased scavenger enzyme catalases (CAT) in muscle cells, a finding corroborated by a gentamicin induced AKI animal model [24].

The reduction in oxidative stress induced by exercise in the gentamicin model [24] is of particular interest, as in this study the exercise regimen was delivered during the recovery phase of AKI as opposed to a preconditioning exercise regime demonstrated by the other animal studies. The exercised animals had 50% higher plasma nitric oxide (NO) levels compared to the rest group despite an increased rate of urinary NO excretion. Gentamicin has previously [52] been shown to cause a decrease in plasma concentrations of NO, leading to vasoconstriction and ischaemia induced nephrotoxic insult [52]. This suggests a mechanism through which the exercise induced increase in NO may alleviate renal injury. The study also highlighted the antioxidative effect of exercise signified through reduced plasma, urine and renal tissue thiobarbituric acid reactive substances (TBARS) together with increases in the antioxidant kidney defences, CAT and glutathione (GSH).

An increase in the anti-oxidative stress factor superoxide dismutase (SOD) has also been demonstrated in the AKI animal model [24]. An increase in SOD has also been shown in humans follwing both acute and long term exercise [53]. This increase is thought to be secondary to an increase in NO [54] which can be increased through exercise [24]. The increase in anti-oxidative enzymes after long-term exercise and attentuation of oxidative stress demonstrate a further potential mechanism through which exercise can reduce the progression from AKI to CKD.

### Autophagy Upregulation

Autophagy is the process by which damaged intracellular components are cleared and pathogens removed. Animal studies, which used IR [23] and gentamicin [24] models to induce AKI, have reported beneficial effects of exercise through the upregulation of these processes. IR results in AKI due to a period of renal hypoxia which stimulates multiple signalling cascades. This is then followed by reoxygenation which amplifies tissue damage through necrosis, apoptosis and inflammation. Gentamicin, a nephrotoxic agent, induces AKI primarily through tubular damage evident through the loss of epithelial cell brush borders resulting in acute tubular necrosis [55]. Both studies indicated reduced kidney damage in exercise groups through reduced creatinine [23], urea [23], proteinuria [23] and increased NO plasma levels [24]. These biomarker data were corroborated by histological observations, where reduced severity of tubular injury [23] and reduced lymphonuclear infiltrates [24] were seen, indicating more advanced renal recovery in the exercise groups. The key mechanisms of exercise induced renal protection highlighted were autophagy [23] and anti-oxidative stress [24]. As mentioned, autophagy plays a key role in cellular homeostasis and is responsible for the degradation and reprocessing of intracellular components to either result in cell death or survival [56, 57]. An in vivo study [57] demonstrated an increase in autophagy induced cell survival in an IR renal injury model however the exact mechanism leading to the autophagy upregulation was not clear. More work is required to investigate the role of changes in autophagy following exercise and its potential as a renal protective mechanism.

**Fig. 1:**
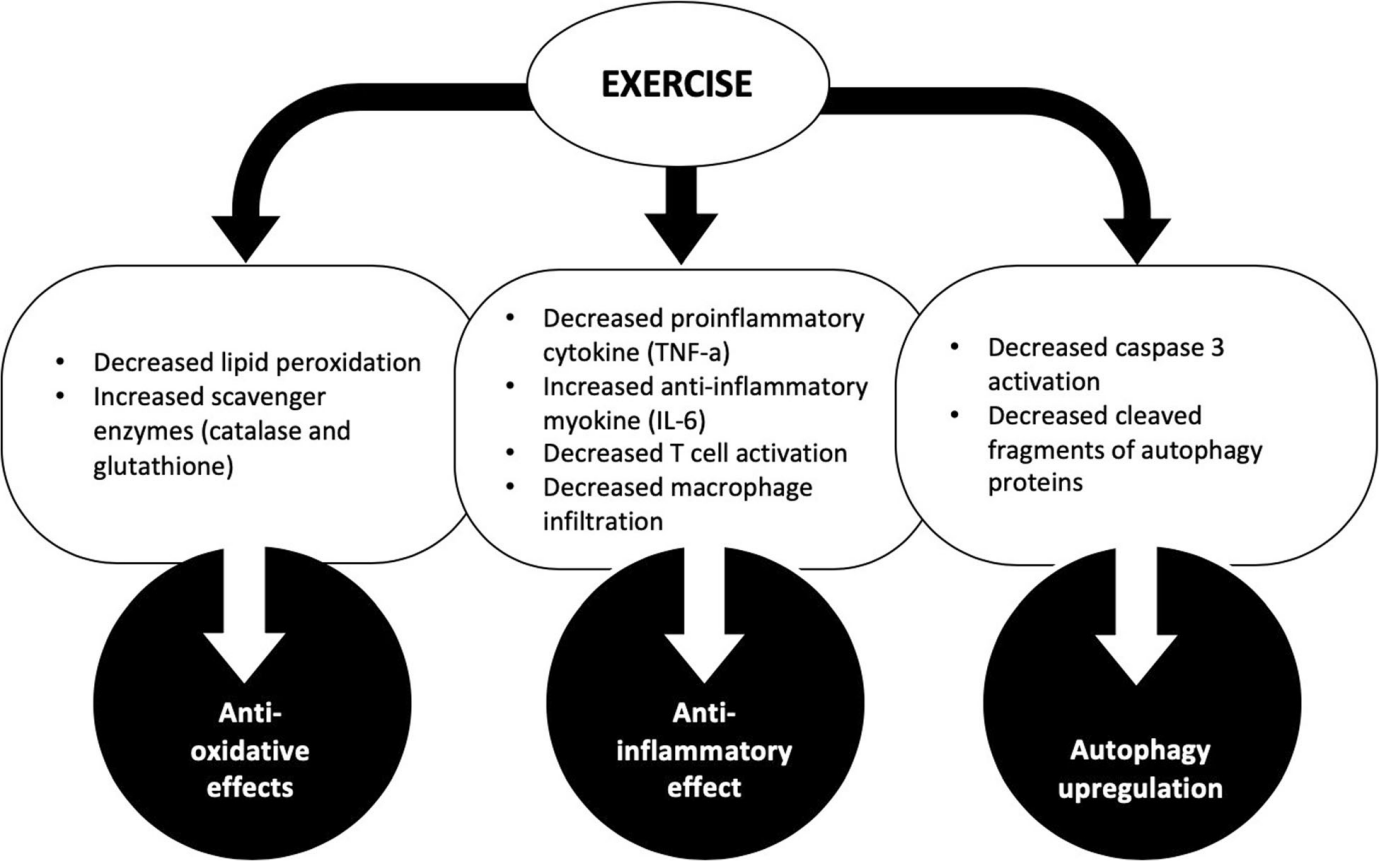
Mechanisms of exercise induced renal protection The different mechanisms through which exercise offers renal protection in AKI animal models. Exercise induces a series of changes within the body (rounded box), ultimately offering renal protection through three main mechanisms (circled)

These animal studies (Table 2) looked at a number of renal biomarkers in order to try and elucidate the mechanisms through which exercise is able to exert this renal protective effect. The main mechanisms identified are highlighted in Fig. 1 with anti-inflammatory and anti-oxidative processes being the most common. The data from animal studies demonstrating the renal protective effects of exercise in both the evolution and recovery of AKI [23–29], highlight the need for clinical trials in this area.

**Table 2:** Animal AKI models

Author, year	Animals	Training programme	Method of inducing AKI	Pre or Post Exercise training?	Results
de Lima [23], 2019	Male Wistar Rats	4-week training programme (5 days a week)	Ischaemia-reperfusion injury	Post	↓blood urea, ↓urinary protein, ↓plasma creatinine, ↓apoptosis, ↓tubular injury, ↓tubular cells degenerative injury, ↑cell regeneration.
Francescato [27], 2018	Male Wistar Rats	4-week (5 days a week; combination of easy and moderate aerobic training	Cisplatin	Post	↓Plasma creatinine, ↓urinary volume, ↓sodium and potassium fractional excretion, ↑urine osmolality, ↓lesions, ↓macrophages, ↓ vimentin, α-sma expression, ↓vascular endothelial growth factor, ↑Nitric oxide
Miyagi [25], 2014	C57Bl6 male mice	6-week training programme	Cisplatin	Post	↓Serum creatinine, ↔apoptosis, ↓TNF-α, ↓IL-10, ↑IL-6, ↔Nrf2, ↑ HO-1.
Miyagi [26], 2018	C57Bl6 male mice,	6-week training programme	Cisplatin	Post	↓Serum creatinine, ↓Kim-1, ↑CD4 + CD69+, ↓CD4 + CD25+, IL-10↓, TNF↓
Oliveira [24], 2017	Male Wistar Rats	30 days of moderate treadmill running (60 min/day for 5 days a week).	Gentamicin (100 mg/kg/day) for 10 days	Pre	↑Urinary, ↓plasma lipid peroxidation, ↓urinary lipid peroxidation, ↓kidney lipid peroxidation, ↑anti-oxidants (catalase & glutathione).
Sossdorf [29], 2013	C57BL/6 N male mice	6 weeks of treadmill running	Sepsis	Pre	↓ creatinine, ↓ BUN, ↓IL-10, ↓ TNF -α, ↓ monocyte chemoattractant protein-1, ↓ NGAL, ↓histological tubular damage
Zeynali [28], 2015	Male Wistar Rats	8-week treadmill training programme (one hour per day/five days per week)	Cisplatin	Post and During	↓Serum blood urea nitrogen, ↓serum creatinine, ↓kidney tissue damage, ↓kidney weight, ↓kidney nitrite, ↓serum nitrite ↓kidney malondialdehyde, ↓serum malondialdehyde

## Future clinical studies

Further work in humans is needed to demonstrate the renal protective effects of exercise that have been observed in animal studies. The need for therapeutic agents to ameliorate renal recovery following AKI in order to reduce the risk of progression to CKD has been highlighted previously [58]. The mechanisms by which exercise could achieve this have been identified in animal studies [23–29]. It is yet to be determined whether the evidence shown in animal studies will also be seen in humans. More specifically whether the anti-inflammatory, anti-fibrotic, anti-oxidative and autophagy upregulation effects seen after exercise in animal AKI models will also be seen in AKI patients. If so, this could pave the way for the development of an exercise regime to ultimately reduce long term renal damage following AKI. Despite the current lack of evidence assessing the efficacy of exercise in the AKI population there is evidence from other diseased populations that may translate.

Exercise also has the ability to impact upon many of the adverse outcomes associated with an episode of AKI. CKD occurs in a large proportion of AKI survivors, and hospital readmission, poor health-related quality of life, cardiovascular events and mortality are common in this patient population [59–61]. A recent report [60] indicated that AKI is associated with both an increased risk of cardiovascular mortality and major cardiovascular events (86 and 38% respectively). Programmes of exercise rehabilitation in cardiovascular patients have been shown to reduce risk of cardiovascular mortality, hospital readmission, health-related quality of life and symptoms of depression [62–64]. Intuitively exercise may have the potential to impact on these adverse outcomes that are associated with AKI and warrants further investigation. Moreover, the suitability of exercise programmes for individuals following a cardiac event suggests that it may also be feasible in the AKI population. This is due to the assumed similarity in functional status of both AKI and cardiac patients since there is a large overlap in these groups with 1 in 4 hospital admitted AKI patients also having a history of ischaemic heart disease [65].

Large observational studies may be initially required to investigate the associations between habitual physical activity levels and renal recovery after AKI. It is possible that higher levels of routine physical activity may protect against AKI and promote renal recovery in the post-AKI period, although this is not clear. If this is indeed the case, then a formal exercise rehabilitation programme may protect against the development of CKD. Similarly, in cardiac and pulmonary disease an exercise rehabilitation programme is effective in protecting against further decline following an acute admission [66, 67]. If such associations exist in people who have developed AKI, a randomised controlled trial (RCT) to test the efficacy of an exercise rehabilitation intervention in the post-AKI recovery period may be appropriate.

## Considerations for the implementation of future clinical studies

### Timing of exercise

There are many different recovery trajectories following AKI, with individuals either having early recovery prior to discharge, late recovery or non-recovery [68]. Observational studies [68, 69] investigating recovery following AKI indicate that recovery continues post discharge with late recovery still clearly associated with better outcomes than non-recovery. Therefore, an exercise intervention would be most effective in the three months following the AKI insult, since renal recovery appears to continue up until this point [70]. Exercise may have the potential to accelerate recovery which could be beneficial, as those with prolonged recovery have worse long term outcomes than individuals for whom recovery is more immediate [71]. An exercise intervention immediately in the post discharge period may be most feasible as this will allow adequate time for patients to at least recover some of their functional status following their acute hospital admission, hence encouraging their participation in the programme.

### Outcomes

**Fig. 2:**
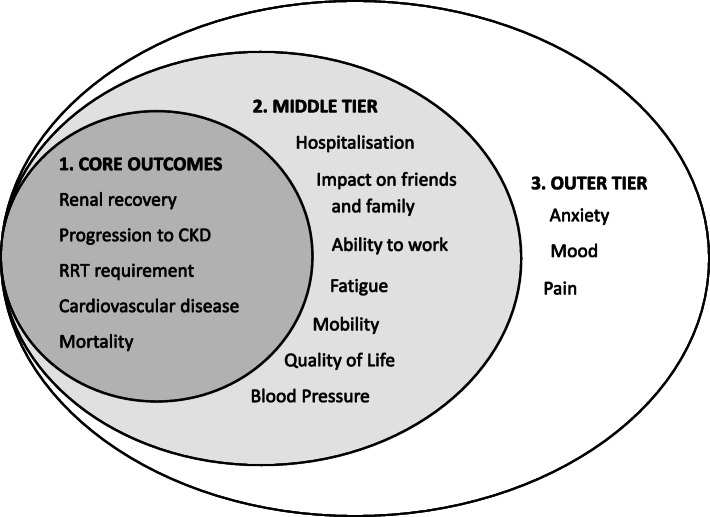
Outcomes for future clinical trials. Figure adapted from the Standardised Outcomes in Nephrology (SONG) core outcomes sets [74]. This figure highlights outcomes of critical importance to people with CKD, which may be used as endpoints in a trial designed to test the effectiveness of exercise interventions following an episode of AKI

Outcomes that are meaningful and relevent to patients (patient-centered) should be used to assess the effectiveness of future exercise interventions. Standardised outcomes for clinical trials have been set out in numerous medical specialities, including nephrology [72]. Currently, however, there are no specific standardised core outcomes for AKI patients. Many outcomes such as health related quality of life and mortality are important to all kidney diseased patient populations and therefore can be translated to the AKI setting. CKD specific outcomes are also still relevant since AKI and CKD are considered on a spectrum as opposed to two separate conditions hence there is a large overlap between patient outcomes [3]. The current standardised outcomes for patients with varying levels of CKD [73] have therefore influenced the outcomes highlighted in Fig. 2 which we believe are important for future trials in the AKI population.

### Setting of exercise delivery

The suitability of the exercise programme to match the patient demographic is essential in ensuring that there is active engagement. Patients age and multimorbidity warrant special attention as it has previously been shown that people with lower physical activity prior to an acute myocardial infarction have greater concerns in participating in exercise rehabilitation programmes afterwards [75]. The type of exercise delivery (e.g. supervised versus home based) also requires investigation to ensure adequate uptake and engagement. Evidence suggest there are no real difference in outcomes including mortality, cardiac events and exercise capacity between supervised and home-based cardiac rehabilitation programmes [76–78]. Despite this, national audit figures for cardiac rehabilitation demonstrated that more than 75% of patients opt for supervised rehabilitation programmes [79] suggesting this may also be the optimum setting for people after AKI. The barriers and enablers to supervised versus home based exercise programmes, along with other important practical points to consider when implementing an exercise intervention, are outlined in Table 3. Supervised cardiac rehabilitation programmes are both effective and well established, however recently studies have shown home based programmes to be similarly effective in improving health-related quality of life and hospital admissions [86] as well as being cost effective [87]. Consequently, it is important to evaluate both the type of programme and the setting in which it should be delivered in order to facilitate maximum adherence.

**Table 3:** Barriers and enablers to current exercise rehabilitation programmes [80–85]

Theme	Barriers	Enablers
Referral to programme	Oversight of patient’s eligibility.	Good relationship between health care practitioner and the patient allows the patient to have understanding of the relevance, importance and benefits of referral to the programme. Automated referral system.
Geographical Factors	Long travel times. Difficulties in accessing transport, especially in rural areas where there is limited public transport available. High costs associated with travel.	Nearby facilities with short travel times. Good support from family and friends who offer assistance with transport.
Psychosocial Factors	Negative health attitude, low level of motivation, underlying mental health issues. Lack of support from friends and family. Lower socioeconomic status resulting in financial strain hence unwilling to take time off work. Patients overwhelmed with information during their hospital stay, contributing to an overall sense of helplessness and low motivation. Linguistic and cultural differences. Unclear of benefits of the programme, poor understanding or disbelief in the positive outcomes. Feeling of embarrassment taking part in such a programme. Patient illness.	Motivation to improve health and feel better. Encouragement from others. Programmes offering psychological support.
Supervised Programme	Lack of staff. Lack of resources. Higher costs (compared to home based). Timings of the programme restricted to weekday working hours.	Presences of programme facilitator during exercise acts as a motivator and reassures patients. Patients held accountable to attend. Group programmes allow participants to interact and share experiences.
Home Based Programme	Reduced intensity of programme. Less support available to patients from health care professionals. Reduced patient adherence and progression. Reduced patient accountability.	Increased flexibility. No transport issues. Potentially lower costs (compared to supervised).

## Conclusion

AKI is a common condition often with a complex and multifactorial aetiology, the mechanisms behind which are poorly understood. More therapeutic options to improve renal recovery after AKI are required [58]. Exercise has been effective in achieving this in animal studies [23–29] through its anti-inflammatory, anti-fibrotic, and anti-oxidative effects as well as through autophagy upregulation. At present, the promising results from animal studies have not been replicated in clinical trials. More work is needed with observational studies initially being conducted to reproduce patterns seen in animal studies followed by well-designed clinical trials to test the effects of an exercise rehabilitation intervention on the core outcomes that we have identified. An exercise rehabilitation programme for AKI patients could ultimately improve renal recovery following AKI, reduce progression to CKD and improve long term patient outcomes.

## Data Availability

The datasets used and/or analysed during the current study are available from the corresponding author on reasonable request.

## Abbreviations

AKI: Acute kidney injury
Bcl2: B-cell lymphoma 2
BAX: Bcl-2 associated X protein
CAT: Catalase
CKD: Chronic kidney disease
ESKD: End stage kidney disease
GFR: Glomerular filtration rate
GSH: Glutathione
ICU: Intensive care unit
IL-6: Interleukin-6
IR: Ischaemia-reperfusion
Kim-1: Kidney injury molecule-1
MDA: Malondialdehyde
NHS: National health service
NO: Nitric oxide
RCT: Randomised controlled trial
SOD: Superoxide dismutase
TNF-a: Tumour necrosis factor-alpha
TBARS: Thiobarbituric acid reactive substances
